# Arts and Ageing; Life Expectancy of Historical Artists in the Low Countries

**DOI:** 10.1371/journal.pone.0082721

**Published:** 2014-01-08

**Authors:** Fereshta Mirzada, Anouk S. Schimberg, Frouke M. Engelaer, Govert E. Bijwaard, David van Bodegom, Rudi G. J. Westendorp, Frans W. A. van Poppel

**Affiliations:** 1 Leyden Academy on Vitality and Ageing, Leiden, the Netherlands; 2 Leiden University Medical Center, Leiden, the Netherlands; 3 Netherlands Interdisciplinary Demographic Institute (NIDI), the Hague, the Netherlands; 4 Department of Sociology, Utrecht University, Utrecht, Netherlands; University Medical Center Rotterdam, The Netherlands

## Abstract

Practising arts has been linked to lowering stress, anxiety and blood pressure. These mechanisms are all known to affect the ageing process. Therefore, we examine the relation between long-term involvement in arts and life expectancy at age 50 (LE50), in a cohort of 12,159 male acoustic, literary and visual artists, who were born between 1700 and 1899 in the Low Countries. We compared the life expectancy at age 50 of the various artists with the elite and middle class of that time. In the birth cohorts before 1850, acoustic (LE50:14.5–19.5) and literary artists (LE50:17.8–20.8) had a similar life expectancy at age 50 compared to the elite (LE50:18.0–19.0). Only visual artists (LE50:15.5–17.1) had a lower life expectancy at age 50 compared to the elite at that time. For the most recent birth cohorts from 1850 through 1899, the comparison between artists and the elite reversed and acoustic and literary artist had a lower life expectancy at age 50, while visual artists enjoyed a similar life expectancy at age 50. Although artists belonged to the middle socioeconomic class and lived predominantly in urban areas with poor living conditions, they had a life expectancy similar to the elite population. This is in line with observed favourable effects of practicing arts on health in the short-term. From our historical analysis, we hypothesize several mechanisms through which artistic creativity could influence the ageing process and life expectancy. These hypotheses, however, should be formally tested before any definite conclusions on effects of arts on ageing can be drawn.

## Introduction

Since ancient times arts have been used for therapeutic purposes to enhance health. Hippocrates played music for his mental patients and Aristotle described music as a force that purified the emotions [1]. In the thirteenth century, Arab hospitals also contained music-rooms for the benefit of the patients [2]. Since that time, colleges and universities developed programs to train musicians how to use music for therapeutic purposes. Also today, acoustic, literary and visual arts are used to improve the health conditions in chronically ill patients [3]-[5]. Current research has shown favourable effects of engaging in arts on health, via several mechanisms such as reducing stress, anxiety, pain and blood pressure or improving the immune response and quality of life [6]. Via such mechanisms, artistic interventions can increase general health, decrease doctor visits, and reduce medication use and improve strategies to cope with chronic diseases [3], [6].

Many of the above described effects of arts on health, are also known to affect the ageing process. High levels of stress, blood pressure or anxiety, have been found to increase the risk of ageing-related diseases, such as cardiovascular disease [7], [8]. In addition, lower stress and anxiety are strongly linked with better well-being, which again is associated with longevity [9]–[14]. It is therefore of interest to study the long term effects of performing arts on life expectancy. Up to now, this has been only studied on the short term, and the effect of practicing arts on ageing and life expectancy remains unknown. One earlier historical study [15] has shown that visual artists enjoyed a life expectancy that was similar to the life expectancy of the elite, but the question remained whether this was due to their artistic profession or due their socioeconomic status or other determinants. No studies have been performed on the life expectancy of other artists.

Therefore, we studied the relation between long-term involvement in arts and life expectancy at age 50, in a unique historical cohort of 12,159 male artists from different disciplines, who were born between 1700 and 1899. By studying different groups and types of artists we gain more insight in whether the observation that visual artists have a similar life expectancy as the elite is a true ‘art-effect’ or an ‘artefact’ of socioeconomic status, selection or other another determinant. This is the first time that the historical life expectancy of different artists is studied in relation to each other and the elite. The aim of this study was to examine ageing-related or senescent mortality, which increases with age more than non-ageing, accident- related mortality, which is also described as background mortality that does not rise with age [16]. A better understanding of the possible favourable effects of engaging in arts on ageing is of great interest, since it could point to possible public health interventions that not only increase the length but also the quality of life.

## Materials and Methods

### Data

We retrieved data from three different databases. Because of the small number of female artists in historical times, we have only studied male artists. Also, because we are interested in ageing-related or senescent mortality and not in child or background mortality, we confined the analysis to people who lived at least till 25 years of age.[16] The Dutch Music Institute (Nederlands Muziek Instituut, The Hague) [17] provided data of 1,543 acoustic artists, comprising both composers (81.7%) and musicians (19.3%). From the total of 1,543 acoustic artists, the sample was reduced by removing the individuals for which the birth year was unknown (125) and additionally those for whom the year of death was unknown (211). Finally, because we place the restriction that the artists should have survived up till age 25, we removed 54 acoustic artist who died before age 26, resulting in a remaining group of 1,153 acoustic artists that we included in our analysis. Next, the Biographic Portal (Biografisch Portaal), a national institute which collects scientific information about leaders and prominent figures from Dutch history, provided a group of 742 literary artists [18]. The group of literary artists consists mainly of poets. From the total of 742 literary artists, the sample was reduced by removing 67 individuals who were born before 1700 and 75 were born after 1899. From the remaining 600 literary artists, we excluded 92 women (we only focus on men). Next, 3 literary artists died before age 26, which resulted in a final sample of 505 literary artists. We derived data of 13,942 visual artists from the RKD-artists database, hosted by the Dutch Institute for Art History (Rijksbureau voor Kunsthistorisch Documentatie, The Hague) [19] This database exists of painters (87,8%) and sculptors (12,2%). For an extensive description of the visual artist data we refer to an earlier study [19]. From the total of 15,419 visual artists Van Poppel et al. [15] used, we removed those who were born before 1700 (3,339) and born after 1899 (1,477) and those who died before age 26 (102). We ended up with a sample of 10,501 visual artists. Finally, we obtained 9,388 elite individuals from the Biographic Portal [18]. This group contained individuals from several occupational fields, including church, education, science, government, nobility and royals, trade and industry, defence forces, judicial system and colonial overseas. The largest subgroup among the elite was from Church, i.e. 2,765 people (31.6%). The majority of all groups consisted of men (78.4–96.4%). From the total of 9,388 elite individuals, we excluded 2,697 who were born before 1700 and 579 who were born after 1899. From the remaining 6,112 elite, we excluded 167 women. From the residual 5,945, 20 died before age 26 and we finally included 5,925 in our analysis.

Historical data on individuals from the middle class were only available for the most recent birth cohorts from 1850–1899. We use data that relate to the country as a whole, thus we can take into account the situation of people living in a variety of ecological, social, and economic circumstances, covering the countryside and small and big towns. For this study we have used data collected in the framework of the Historical Sample of the Netherlands (HSN). From the total of 3,996 individuals in that sample, 1,101 were classified as being middle class. Of these individuals 24 died before age 26 and hence we included 1,077 people in our analysis. For a detailed description of these data and how the socioeconomic status was assessed we refer to an earlier publication by Schenk & van Poppel [20].

All databases are available on request. The birth cohorts covered by these databases lived from the 18^th^ century until the end of the 1900s. Every individual is only once assigned to a group in the database. Hence, there is no overlap between the groups. It is possible that some elite people also practiced arts occasionally, but if such an elite person practiced arts more professionally, he would have been in the artist-database.

By studying the life expectancy at age 50, we have tried to reduce the effect of background mortality, such as early deaths due to child disease, and at middle age and other non-ageing related diseases. Additionally, by studying life expectancy at age 50, we overcome a survivor treatment selection bias, also known as ‘immortal time bias’. In the case of artists for instance, they have to survive up to a certain age in order to become successful and famous artist. A recent example from the literature is a study that suggested a survival advantage for Oscar winners, which appeared to be attributable to ‘immortal time bias’ [21], [22].

### Statistical analysis

We carried out separate analyses by four different birth cohort groups: people born between 1700 to 1749, born between 1750 to 1799, born between 1800 to 1849, and born between 1800 to 1899. The data were analyzed using Gompertz hazard models for each discipline and cohort separately. Based on the estimated model we calculated the implied life-expectancy. For the visual artists we sometimes knew only an interval, sometimes 10 years wide, in which they were born and/or died. We used a previously published method to account for this interval censoring [23].

In analyses of human mortality a Gompertz distribution [23] is known to fit well [24]–[26]. This distribution has two parameters, a shape α and a scale parameter β. The hazard rate, the force of mortality, at age *t* increases exponentially over the life span, 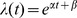. The density, *f*, and the survival, *S*, for a duration *t*, in a Gompertz distribution are:
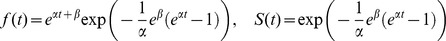
Based on the observed years of birth and death we calculated the implied age of death. For the visual artists we sometimes knew only an interval, sometimes 10 years wide, in which they were born and/or died. In those situations a *minimum* length of life and a *maximum* length of life could be derived. When the date of birth was known exactly but the death was only recorded as occurring within a given interval the *minimum* length of life was calculated as the difference between the date of birth and the earliest date of death reported, and the *maximum* length of life as the difference between the date of birth and the latest date of death. When the date of death was known exactly but the date of birth was recorded as occurring within a specific time-interval, the *minimum* length of life was calculated as the period from the last possible date of birth until the date of death and the *maximum* length of life as the period from the earliest possible date of birth until the date of death. Finally, when both the date of birth and the date of death were known only approximately and both were recorded as falling within particular time intervals. In such cases, the *minimum* length of life was calculated as the period from the latest date of birth until the earliest date of death, and the *maximum* length from the earliest date of birth until the latest date of death. In Van Poppel et al. [15] the procedure to account for this type of interval censoring are discussed in detail.

In addition, by making it a condition that an individual must have already survived to the age of 25, it is possible to take into account the fact that, to be recognised as an artist, an individual needs to have lived long enough to have produced a notable piece of art.

To estimate the parameters of the model we maximized the joint likelihood, which is the product of the individual likelihood contributions. The individual likelihood contribution is the density for those artists whose exact date of birth and date of death were known. For the visual artists we accounted for the interval censoring described above, see [19] or the appendix. Based on the estimated parameters the life expectancy, *LE_50_*, at age 50 can be approximated very well [26].
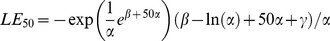
where γ≈0.57722 is the Euler-Mascheroni constant. The variance of the life expectancy at age 50 can be approximated using the delta-method, the standard method in econometrics to obtain the distribution of a nonlinear combination of parameters [27]. Using the estimated *LE_50_*'s and their variance we calculated, for each cohort period and for each of the three artist-groups, the difference of the artist life expectancy with the life expectancy of the elite group (assuming independence the variance of the difference is the sum of the variances).

## Results

We included 12,159 male artists from three different disciplines who were born between 1700 and 1899. The numbers are summarized in table 1. In total 1,153 acoustic, 505 literary and 10,501 visual artists were included. The elite group consisted of 5,925 individuals, born in the same time period. The majority of the individuals (32–69%) were born between 1850 and 1899. Historical data on people from the middle class were only available for the most recent birth cohorts, born after 1850. In total we studied 1,077 individuals from the middle class of that time.

**Table 1:** Characteristics of study population.

	Acoustic artists	Literary artists	Visual artists	Elite	Middle class
**Total n(%)**	1,153 (100)	505 (100)	10,501 (100)	5,925 (100)	1,077 (100)
**Year of birth n(%)**					
**1700–1749**	38 (3)	17 (3)	681 (6)	940 (16)	
**1750–1799**	72 (6)	45 (9)	1,303 (12)	1,424 (24)	
**1800–1849**	243 (21)	130 (26)	2,797 (27)	1,676 (28)	
**1850–1899**	800 (69)	313 (62)	5,720 (54)	1,885 (32)	1,077 (100)

**Figure 1:**
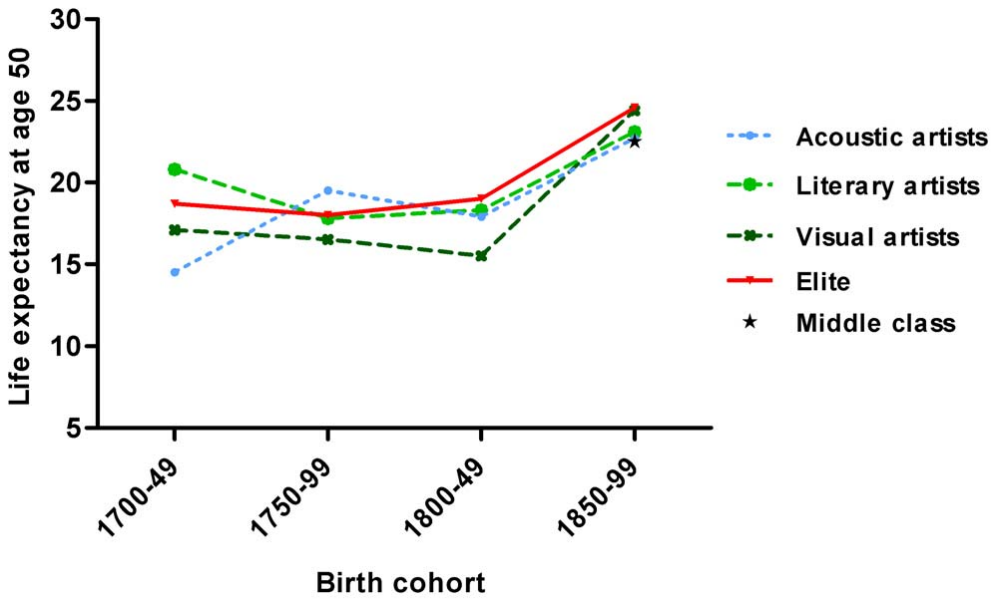
Life expectancy at age 50 of male artists (acoustic, literary and visual), the elite born between 1700 and 1899 and the middle class born between 1850–1899.

We analysed the life expectancy at age 50 from the various groups of artist and first compared their life expectancy to that of the elite. Figure 1 shows the life expectancy at age 50 for the birth cohorts from 1700 to 1749, 1750 to 1799, 1800 to 1849 and 1850 to 1899. From 1700 to 1899, there has been an increase in life expectancy in all groups. For the middle class, data were only available for the most recent studied cohorts, in the figure this point estimate is shown as a *star*.

Next, we tested whether the life expectancy of the artists was different from that of the elite. Table 2 shows the life expectancies at age 50 of artists and elite, the standard errors and tests of significance. Before 1850 artists had a similar life expectancy at age 50 when compared to the elite of that time, except for the visual artists, who had a significant lower life expectancy at age 50. In the most recent period from 1850 to 1899, this pattern reversed and acoustic and literary artists had a significant lower life expectancy at age 50 than the elite. Visual artists and the elite had a similar life expectancy at age 50 of almost 25 years after 1850. Only for the latest period, data of a middle class population were available. During that period, the middle class had an average life expectancy at age 50 of 22.6 years. The various artists had a higher life expectancy than the middle class at that time, although this reached only significance in the largest group of visual artists.

**Table 2:** Life expectancy at age 50 (LE50) of artists compared to the life expectancy at age 50 of the elite, who were born between 1700–1899 and to the life expectancy at age 50 of the middle class who were born between 1850–1899.

Year of birth	Acoustic artists		Literary artists		Visual artists		Elite		Middle class	
	LE50 (SE)	P	LE50 (SE)	P	LE50 (SE)	P	LE50 (SE)	P	LE50 (SE)	P
**1700–1749**	14.5 (2.9)	0.07	20.8 (2.1)	0.17	17.1 (0.6)	0.02	18.7 (0.5)	ref.		
**1750–1799**	19.5 (1.4)	0.16	17.8 (2.1)	0.46	16.5 (0.5)	<0.05	18.0 (0.4)	ref.		
**1800–1849**	17.9 (1.0)	0.14	18.3 (1.4)	0.31	15.5 (0.4)	<0.001	19.0 (0.3)	ref.		
**1850–1899**	22.7 (0.6)	<0.05	23.1 (0.7)	0.02	24.4 (0.2)	0.23	24.6 (0.3)	ref.		
**1850–1899**	22.7 (0.6)	0.43	23.1 (0.7)	0.26	24.4 (0.2)	<0.001	24.6 (0.3)	<0.001	22.6 (0.4)	ref.

## Discussion

Between 1700 and 1849 acoustic and literary artists had a similar life expectancy at age 50, when compared to the elite population of the Low Countries. Only visual artists had a lower life expectancy at age 50 in these birth cohorts. This is striking, since artists belonged predominantly to the middle socioeconomic class [15], [28]. In cohorts born after 1849 acoustic and literary artists had a lower life expectancy compared to the elite, while visual artists enjoyed a similar life expectancy when compared to the elite. Unfortunately, historical data on the middle class population has not been documented. Only for the most recent birth cohorts we have found data, which showed that artists lived longer than the middle class, but this reached only significance in the largest group of visual artists.

Our study differs from a previous study, in which artists were found to have a lower survival compared to popes [29]. Others have shown that visual artists had a relatively high life expectancy, when compared to the elite of their time, which is in line with our observations [15]. We studied different groups and types of artists and found that not only visual, but also, other artists had a similar life expectancy as the elite. Although there are differences between the group sizes of the various artist types, this is the first time that the historical life expectancy of different artists is studied in relation to each other and the elite population. However, whether the observed differences are related to the practicing of art or due to socioeconomic status, selection effects or other characteristics of artists remains to be further studied.

During the studied period from 1700 to 1899, life expectancy increased for both artists and the elite. Possible mechanisms that have driven this increase in life expectancy could be improvements in living conditions, better hygiene and public health [30]. From 1850 onwards, with the epidemiologic transition, the increase in life expectancy is known to be especially driven by improvements in wealth and public health [30]. Up to 1850, visual artists were the only artists having a lower life expectancy than the elite in our study. Earlier it has been shown that painters have a lower life expectancy than sculptors, which was attributed among other factors to differences in exposure to toxic materials [15], [31]. This could partly explain the observed lower life expectancy of visual artists in our study, since the visual artist group consisted primarily of painters (81.7%). Additionally, it is possible that other factors, such as differences in terms of educational level, socioeconomic status or familial background could be behind the difference in life expectancy between visual artists and other artists groups.

It is tempting to hypothesize about possible mechanisms behind our findings. Socioeconomic status is strongly linked to life expectancy [32]. Even today, rich people outlive poor people by seven years in the Netherlands [33]. How socioeconomic status was related to life expectancy in the past is however debated [34]. Historical data suggest that socioeconomic status was not linked to life expectancy as today [35]. By contrast, contemporary data from a rural population in Ghana, have shown that even under those adverse conditions, individuals with a higher socioeconomic status have a considerable survival advantage [36]. In our dataset, there is no information on the socioeconomic status of the individual artists. Little is known in general about the socioeconomic status of artists in historical times, but prior to the industrialization artists usually descended from middle class families [15], [28]. Detailed studies on the socio-economic position of visual artists in the first half of the nineteenth century in the cities of Dordrecht and The Hague, have confirmed that the majority of the artists were able to live a rather comfortable life; the most successful were able to earn an income that was higher than that of the middle classes (artisans, merchants, public servants) they originated from [37]. This picture is confirmed by the outcome of a study on the socio-economic position of the visual artists in the second half of the same century in The Hague and Amsterdam. It would seem that in this period the visual artists were quite successful in climbing the social ladder [38]. In the 18^th^ century, the income of musicians was not sufficient to make a living. Some musicians therefore, additionally worked in other professions too; in our study 34% of the acoustic artists were having another profession next to their artistic career. Literary artists also belonged to the middle class in economical terms. Only from the end of the 19^th^ century onwards, by industrialization of book printing techniques, their socioeconomic status improved [39].

The low life expectancy among the middle and lower class has been related to poor sanitation, malnutrition, overcrowding, polluted water, less access to health care and living in unsafe and unhealthy conditions [40]. It is important in this respect that most artists lived in urban areas where their audience and clients lived. Here, poor sanitary conditions and epidemics of infectious diseases used to cause high mortality [15]. This makes it especially striking that the life expectancy of most artists was similar compared to the higher classes with much better living circumstances. After the industrialization, the mass production of cheaper products led to an increased access to musical instruments for people of lower classes. Additionally, living conditions in the cities deteriorated and these processes could have contributed to the lower life expectancy of artists compared to the elite born in the period after 1850 until 1900 [41].

Others have studied several mechanisms through which artistic creativity could have an effect on health and ageing [42]–[55]. Some have found that art and music interventions can enhance bodily control or improve pain management [43]. In addition, it reduces stress and anxiety with consequent effects upon heart rate, respiration, blood pressure, brain function and immune response [44]. Singing for instance, has been found to increase certain chemicals, such as endorphins (natural pain killers) and immunoglobulin A (immune function), which enhances the pulmonary work out, oxygen intake and increases circulation [45]. Moreover, music distracts one from feelings of illness, gives patients sense of control and lowers their blood pressure [43]–[45]. Also, music has been found to boost the immune function [46], increase cognitive function [47] and improve memory performance [48], concentration and attention [48]. Music increases physical performance by increasing psychological arousal, reduces feelings of fatigue and improves motor coordination [49], [50]. Studies on visual arts revealed similar results, such as reducing anxiety, improving vital signs, diminished cortisol levels related to stress and improved sleeping patterns [51], [52]. In addition, creative writing such as poetry has been found to have beneficial psychological effects on coping mechanisms for depression and increases the likelihood that patients can stop their antidepressants [53]. Finally, it can boost the immune system and improve pain control [54], [55].

We analysed a unique cohort with artists from three different disciplines over a long time span from 1700 till the end of the next century. The Low Countries form an interesting case to study the effect of arts because of their rich tradition in artistic creativity [15]. A limitation of our study is that the historical records contained very few women and hence, we have only studied the life expectancy in men. Furthermore, it is possible that our database contains only the artists who survived long enough to have the opportunity to become well known and for that reason also well documented. In this case our observations would be partly explained by survivor treatment selection bias. However, we have tried to overcome this problem by studying life expectancy at age 50.

All in all, we have found that in cohorts born before 1850, acoustic and literary artists had comparable life expectancy as elite groups, despite belonging to middle socioeconomic class and living in urban areas with poor living conditions. Only visual artists had a lower life expectancy compared to the elite. From our historical analysis, we hypothesize several mechanisms through which artistic creativity could influence ageing and life expectancy. These hypotheses, however, should be formally tested before any definite conclusions about the effects of arts on ageing can be drawn.

## References

[1] Misic P, Arandjelovic D, Stanojkovic S, Vladejic S, Mladenovic J (2010) Music therapy. Eur Psychiatry 25: 839.

[2] Antrim DK (1944) Music Therapy. The musical quarterly 30: 409.

[3] Kelly CG, Cudney S, Weinert C (2012) Use of creative arts as a complementary therapy by rural women coping with chronic illness. J Holist Nurs 30: 48–54. 22024956 10.1177/0898010111423418

[4] Makin S, Gask L (2012) ‘Getting back to normal’: the added value of an art-based programme in promoting ‘recovery’ for common but chronic mental health problems. Chronic Illness 8: 64–75. 21985790 10.1177/1742395311422613

[5] Nanda U, Eisen S, Zadeh RS, Owen D (2011) Effect of visual art on patient anxiety and agitation in a mental health facility and implications for the business case. J Psychiatr Ment Health Nurs 18: 386–393. 21539683 10.1111/j.1365-2850.2010.01682.x

[6] Cohen GD (2006) Research on creativity and aging: The positive impact of the arts on health and illness. Generations 30: 7–15.

[7] Steptoe A, Brydon L (2009) Emotional triggering of cardiac events. Neurosci Biobehav Rev 33: 63–70. 18534677 10.1016/j.neubiorev.2008.04.010

[8] Merz CNB, Dwyer J, Nordstrom CK, Walton KG, Salerno JW, et al (2002) Psychosocial stress and cardiovascular disease: pathophysiological links. Behav Med 27: 141–147. 12165968 10.1080/08964280209596039 PMC2979339

[9] Danner DD, Snowdon DA, Friesen WV (2001) Positive emotions in early life and longevity: findings from the nun study. J Pers Soc Psychol 80: 804–813. 11374751

[10] Davidson KW, Mostofsky E, Whang W (2010) Don't worry, be happy: positive affect and reduced 10-year incident coronary heart diseases: The Canadian Nova Scotia Health Survey. Eur Heart J 31: 1065–1070. 20164244 10.1093/eurheartj/ehp603 PMC2862179

[11] Diener E, Chan MY (2011) Happy people live longer: subjective well-being contributes to health and longevity. Appl Psychol Health Well-being 3: 1–43.

[12] Chida Y, Steptoe A (2008) Positive psychological well-being and mortality: a quantitate review of prospective observational studies. Psychosom Med 70: 741–756. 18725425 10.1097/PSY.0b013e31818105ba

[13] Howell RT, Kern ML, Lyubomirsky S (2007) Health benefits: meta-analytically determining the impact of well-being on objective health outcomes. Health Psychol Rev 1: 83–136.

[14] Perenboom RJM, Van Herten LM, Boshuizen HC, Van Den Bos GAM (2004) Trends in life expectancy in wellbeing. Soc Indic Res 65: 227–244.

[15] Van Poppel FWA, van de Kaa DJ, Bijwaard GE (2013) Life expectancy of artists in the Low Countries from the fifteenth to the twentieth century. Pop Stud-J Demog 67 3: 275–292 DOI:10.1080/00324728.2013.765955 10.1080/00324728.2013.765955 23432180

[16] Bongaarts J (2005) Long-Range trends in adult mortality: models and projection methods. Demogaphy 42 1: 23–49. 10.1353/dem.2005.0003 15782894

[17] Nederlands Muziek Instituut (NMI). Available: http://www.nederlandsmuziekinstituut.nl/. Accessed: 1 May 2012

[18] Biografisch Portaal. Available: http://www.biografischportaal.nl/en/. Accessed: 1 Oct 2012.

[19] Rijksbureau voor Kunsthistorische Documentatie or RKD (Den Haag). Available: http://english.rkd.nl/Databases/RKDartists. Accessed: 15 May 2011.

[20] Schenk N, van Poppel FWA (2011) Social class, social mobility and mortality in the Netherlands, 1850–2004. Explorations in Economic History 48: 401–417.

[21] Redelmeier DA, Singh SM (2001) Survival in Academy Award-winning actors and actresses. Ann Intern Med 134: 955–962. 11352696 10.7326/0003-4819-134-10-200105150-00009

[22] Sylvestre MP, Huszti E, Hanley JA (2006) Do Oscar winners live longer than less successful peers? A reanalysis of the evidence. Ann Intern Med 145: 361–363. 16954361 10.7326/0003-4819-145-5-200609050-00009

[23] Gompertz B (1825) On the nature of a function expressive of the law of human mortality, and on a new mode of determining the value of life contingencies,. Philosophical Transactions of the Royal Society 115: 513–585. 10.1098/rstb.2014.0379 PMC4360127 25750242

[24] Gavrilov LA, Gavrilova NS (1991) The Biology of Life Span: A Quantitative Approach. Chur, Switzerland: Harwood.

[25] Heligman L, Pollard JH (1980) The age pattern of mortality,. Journal of the Institute of Actuaries 107: 49–80.

[26] Lenart A (2012) The Moments of the Gompertz distribution and the maximum likelihood estimation of its parameters,. Scandinavian Actuarial Journal 1–23 DOI:1080/03461238.2012.687697

[27] Cameron AC, Trivedi PK (2005) Microeconometrics: Methods and Applications. Cambridge, USA: Cambridge UP.

[28] Salmen W, Kaufman H, Reisner B (1983) The Social Status of the Professional Musician from the Middle Ages to the 19th Century, Pendragon.

[29] Carrieri MP, Serraino D (2005) Longevity of popes and artists between the 13th and the 19th century. Int J Epidemiol 34: 1435–1444. 16260451 10.1093/ije/dyi211

[30] Omran AR (1971) The epidemiologic transition: a theory of the epidemiology op population change. Milbank Mem Fund Q 49 4: 509–538. 5155251

[31] Dobson R (2007) Did sculpting give artists a health advantage before antibiotics? Brit Med J 335: 1233.

[32] Marmot M (2005) Social determinants of health inequalities. Lancet 365: 1099–1104. 15781105 10.1016/S0140-6736(05)71146-6

[33] Centraal Bureau voor de Statistiek (CBS), Levensverwachting naar inkomen, gemiddelde over 2004–2007. Available: http://www.cbs.nl/nl-NL/menu/home/default.htm. Accessed: 15 Jan 2013.

[34] Birchenall JA (2007) Economic development and the escape from high mortality. World development 35: 543–568.

[35] Bengtsson T, van Poppel F (2011) Socioeconomic inequalities in death from past to present: an introduction. Explorations in economic History 48: 343–356.

[36] Van Bodegom D, Rozing MP, May L, Meij HJ, Thomése F, et al (2013) Socio-economic status determines sex-dependent survival of human offspring. Evol Med Public Health 1: 37–43. 10.1093/emph/eot002 PMC3868360 24481185

[37] Hoogenboom A (1993) De stand des kunstenaars. Den Haag: Primavera Pers 97–127.

[38] Stolwijk C (1998) Uit de schilderswereld. Primavera Pers 250–272.

[39] Van Lente D (1996) Drukpersen, papiermachines en lezerspubliek: de verhouding tussen technische en culturele ontwikkelingen in Nederland in de negentiende eeuw. Nijmegen: SUN. 246–263.

[40] Haines MR (2004) Growing incomes, shrinking people—can economic development be hazardous to your health? Historical evidence for the United States, England, and the Netherlands in the nineteenth century. Soc Sci Hist 28: 249–270.

[41] Birchenall JA (2007) Economic development and the escape from high mortality. World development 35: 543–568.

[42] Brodzinski E, Munt D (2009) Examining creativity in health and care. Health Care Anal 17: 277–284. 19784780 10.1007/s10728-009-0128-x

[43] Cohen GD (2009) New theories and research findings on the positive influence of music and art on health with ageing. Arts & Health 1: 48–62.

[44] Teng XF, Wong MYM, Zhang YT (2007) The effect of music on hypertensive patients. Conf Proc IEEE Eng Med Biol Soc 4649–4651. 18003042 10.1109/IEMBS.2007.4353376

[45] Bernardi L, Porta C, Sleight P (2006) Cardiovascular, cerebrovascular, and respiratory changes induced by different types of music in musicians and non-musicians: the importance of silence. Heart 92: 445–452. 16199412 10.1136/hrt.2005.064600 PMC1860846

[46] Kuhn D (2002) The effects of active and passive participation in musical activity on the immune system as measured by salivary immunoglobulin A (SIgA). J Music Ther 39: 30–39. 12015810 10.1093/jmt/39.1.30

[47] Mammarella N, Fairfield B, Cornoldi C (2007) Does music enhance cognitive performance in healthy older adults? The Vivaldi effect. Aging Clin Exp Res 19: 394–399. 18007118 10.1007/BF03324720

[48] Patston LL, Hogg SL, Tippett LJ (2007) Attention in musicians is more bilateral than in non-musicians. Laterality 12: 262–272. 17454575 10.1080/13576500701251981

[49] Bernatzky G, Bernatzky P, Hesse HP, Staffen W, Ladurner G (2004) Stimulating music increases motor coordination in patients afflicted with Morbus Parkinson. Neurosci Lett 361: 4–8. 15135879 10.1016/j.neulet.2003.12.022

[50] Rosenkranz K, Williamon A, Rothwell JC (2007) Motorcortical excitability and synaptic plasticity is enhanced in professional musicians. J Neurosci 27 19: 5200–5206. 17494706 10.1523/JNEUROSCI.0836-07.2007 PMC6672373

[51] Reynolds F, Prior S (2003) A lifestyle coat-hanger: a phenomenological study of the meanings of artwork for women coping with chronic illness and disability. Disabil Rehabil 25: 785–794. 12959359 10.1080/0963828031000093486

[52] Collie K, Bottorff J, Long BC (2006) A narrative view of art therapy and art making by women with breast cancer. J Health Psychol 11: 761–775. 16908471 10.1177/1359105306066632

[53] Pennebaker JW (1997) Writing about emotional experiences as a therapeutic process. Psychol Sci 8: 162–166.

[54] Esterling BA, L'Abate L, Murray EJ, Pennebaker JW (1999) Empirical foundations for writing in prevention and psychotherapy: mental and physical health outcomes. Clin Psychol Rev 19: 79–96. 9987585 10.1016/s0272-7358(98)00015-4

[55] Petrie KJ, Fontanilla I, Thomas MG, Booth RJ, Pennebaker JW (2004) Effect of written emotional expression on immune function in patients with human immunodeficiency virus infection: a randomized trial. Psychosom Med 66: 272–275. 15039514 10.1097/01.psy.0000116782.49850.d3

